# Highlight: The Epigenetics of Life at 12,000 ft

**DOI:** 10.1093/gbe/evaa266

**Published:** 2021-02-04

**Authors:** Casey McGrath

Humans inhabit an incredible range of environments across the globe, from arid deserts to frozen tundra, tropical rainforests, and some of the highest peaks on Earth. Indigenous populations that have lived in these extreme environments for thousands of years have adapted to confront the unique challenges that they present. Approximately 2% of people worldwide live permanently at high altitudes of over 2,500 m (1.5 miles), where oxygen is sparse, UV radiation is high, and temperatures are low. Native Andeans, Tibetans, Mongolians, and Ethiopians exhibit adaptations that improve their ability to survive such conditions. Andeans, for example, display increased chest circumference, elevated oxygen saturation, and a low hypoxic ventilatory response, enabling them to thrive at exceptionally high elevations. Although it is clear that there is a genetic component to these adaptations, exposure to high altitudes during early development is also known to play a role, although the underlying mechanism for this remains poorly understood. In a new study in *Genome Biology and Evolution* titled “Genome-Wide Epigenetic Signatures of Adaptive Developmental Plasticity in the Andes,” Ainash Childebayeva, a doctoral student at the University of Michigan at the time of the study, and her colleagues sought to answer this question by studying members of the Peruvian Quechua, who live at high altitudes in the Andes. Their work reveals that mechanisms like DNA methylation may be involved in adaptation to high altitudes, and their findings have potential implications for the long-term health of those living at such heights (Childebayeva et al. 2021).

Adaptations are typically thought of as genetic changes leading to the manifestation of a certain physiological trait, or phenotype. In a phenomenon known as developmental adaptation or adaptive plasticity, however, a certain genetic background merely serves as the prerequisite, and exposure to a certain environmental stimulus—generally during early development—is further required for the trait to be expressed. According to Childebayeva and co-authors, “There are several examples of Andean high-altitude adaptive phenotypes where developmental adaptation plays a key role in the manifestation of the adult phenotype.” For example, Andeans who are lifelong residents of high altitude display greater lung volumes than those of Andean ancestry who were born and raised at sea level.

To reveal the biological mechanisms enabling this interplay between environment, development, and genetics, Childebayeva and her collaborators focused on epigenetics, the study of modifications that alter the DNA molecule without changing the order of nucleotides. Methylation is one type of epigenetic mark in which a methyl group is added to the cytosine nucleotides contained in DNA. Methylation suppresses the transcription of associated genes, thereby influencing an organism’s biology by regulating protein expression. Importantly, DNA methylation patterns are established prenatally and in the early postnatal period, after which they remain relatively stable, providing an early developmental window during which environmental exposures may help shape an individual’s phenotype.

**Fig. 1:**
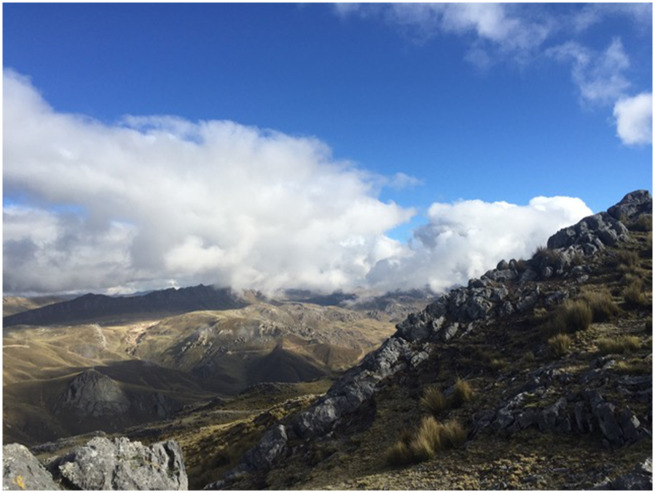
Field site at Cerro de Pasco, Peru. Credit: Abigail Bigham.

The Quechua, an indigenous group native to Peru, have lived on the Andean Altiplano at an average elevation of 12,000 ft (over 3,600 m) for 11,000 years (fig. 1). In order to investigate the potential role of epigenetics in developmental adaptation to high altitudes, the study’s authors evaluated DNA methylation patterns across the genome in three groups of Peruvian Quechua with different altitude exposures: high-altitude Quechua, who had lifetime exposure to high altitude; migrant Quechua, who were born at high altitude but subsequently moved to low altitudes; and low-altitude Quechua, who were lifelong residents of low altitude, despite the fact that their parents and both sets of grandparents were of highland Quechua ancestry. By comparing which DNA positions were methylated in high-altitude and migrant Quechua, who shared early childhood exposure to high altitudes, with those methylated in low-altitude Quechua, who shared ancestry but were not exposed in childhood, the authors were able to untangle the effects of developmental exposure to altitude and genetics.

The study identified specific positions and regions of DNA in which methylation was associated with either lifelong or early altitude exposure. Some of these regions were associated with genes previously linked to high-altitude adaptation, such as those involved in red blood cell production, glucose metabolism, and skeletal muscle development. In particular, some of these genes have been previously implicated in scans of genetic adaptations in other high-altitude populations, such as Tibetans and Mongolians, indicating that both genetic and epigenetic mechanisms may be acting on similar pathways in multiple high-altitude groups. These findings support the idea that epigenetics are involved in developmental adaptation and that early developmental exposures can have persistent impacts on DNA methylation patterns.

The study’s findings also have implications for the health of high-altitude populations. For example, two of the methylated regions associated with high altitude overlapped genes linked to idiopathic pulmonary fibrosis, a condition characterized by irreversible fibrosis of the lung that is known to be associated with hypoxia (lack of oxygen). This suggests that high-altitude populations may have different susceptibilities to this condition or distinct pathological features compared with low-altitude populations. Moreover, the authors estimated the “epigenetic age” of the three groups of Quechua, a phenomenon that reflects the state of the epigenetic maintenance system and can serve as a marker of premature biological aging. They found that those with lifelong exposure to high altitude showed accelerated epigenetic aging compared with those who were lifelong residents of low altitude, likely reflecting the strain that hypoxia places on the cellular machinery.

According to Childebayeva, the resources and collaborators at the Cerro de Pasco High-Altitude laboratory, associated with the Cayetano Heredia University in Lima, Peru, were key to the completion of this project. Researchers have been studying high-altitude adaptation in Cerro de Pasco for almost one hundred years, with one of the first studies taking place in 1921–1922 (Barcroft et al. 1923). Now in the Department of Archaeogenetics at the Max Planck Institute for the Study of Human History, Childebayeva hopes to one day extend this work by studying developmental adaptation in the high-altitude populations of Central Asia, shedding further light on the epigenetic mechanisms that allow humans to push the upper limits of high-altitude habitation.
